# Validation of the Personality Disorder Severity for ICD‐11 (PDS‐ICD‐11) Scale in a Danish Mixed Clinical and Prison Treatment Sample

**DOI:** 10.1002/pmh.70039

**Published:** 2025-09-16

**Authors:** Rachael Martin, Martin Sellbom, Bo Bach

**Affiliations:** ^1^ School of Psychological Sciences Monash University Melbourne Australia; ^2^ Psychiatric Research Unit Center for Personality Disorder Research, Region Zealand Slagelse Denmark

**Keywords:** forensic, PDS‐ICD‐11, personality disorder, severity, substance use

## Abstract

The International Classification of Diseases, 11th Edition (ICD‐11) has adopted a new model for the diagnosis of personality disorder (PD), and novel measures have therefore been developed to assist in its assessment. This study examined the psychometric properties of the Personality Disorder Severity ICD‐11 (PDS‐ICD‐11) scale in a mixed sample of Danish adults in prison (*n* = 101; 100% males; M_age_ = 34.57, SD_age_ = 9.27) or in a rehabilitation program (*n* = 132; 64% males; M_age_ = 39.47, SD_age_ = 11.60) with co‐occurring substance use dependency and personality pathology. Participants were administered the PDS‐ICD‐11, Level of Personality Functioning Scale—Brief Form 2.0 (LPFS 2.0), World Health Organization Well‐being Index (WHO‐5), Symptom Checklist‐90‐Revised (SCL‐90‐R), Personality Inventory for DSM‐5 Short Form (PID‐5‐SF) and the Schema Mode Inventory (SMI). The PDS‐ICD‐11 items conformed to a unidimensional structure, and the total score demonstrated relevant associations with severity indexes, maladaptive personality traits, well‐being and dysfunctional schema modes. The PDS‐ICD‐11 was similarly correlated with both internalising and externalising measures of personality dysfunction across the sample, with the incarcerated sample demonstrating stronger associations with externalising personality traits. Overall, these findings support the validity and utility of the PDS‐ICD‐11 for the assessment of ICD‐11 PD in externalising forensic and rehabilitation samples.

The classification of Personality Disorders (PDs) in the ICD‐11 moves away from traditional categorical diagnoses (e.g., antisocial PD and borderline PD) and instead adopts one general PD diagnosis specified by personality dysfunction (i.e., none, personality difficulty, mild PD, moderate PD and severe PD). Personality dysfunction considers a person's self‐ and interpersonal functioning, their emotional, cognitive and behavioural manifestations of personality disturbance, global distress and functional impacts in occupational, social and personal domains. Although it is not the focus of the current paper, following the assessment of personality severity, clinicians may opt to further specify up to five trait domains (Negative Affectivity, Detachment, Dissociality, Disinhibition and Anankastia) and a borderline pattern specifier as needed (World Health Organisation [WHO] 2024). The ICD‐11 PD severity dimension is similar to Criterion A of the Alternative Model for Personality Disorders (AMPD) in the Diagnostic and Statistical Manual of Mental Disorders, 5th edition (DSM‐5; American Psychiatric Association [APA] 2013); however, it diverges in its explicit consideration of psychosocial impairment and distress, and discrete emotional, cognitive and behavioural manifestations of personality disturbance. While existing measures for the AMPD can be used to assess PD severity (e.g., the Level of Personality Functioning Scale ‐ Brief Form; Bach and Hutsebaut 2018), they do not align with the ICD‐11 model directly (Bach et al. 2021). Since its development in 2021, the Personality Disorder Severity Scale ICD‐11 (PDS‐ICD‐11) has therefore been the primary measure of ICD‐11 PD severity (Bach et al. 2021). Both self‐ and clinician‐report forms are available for the PDS‐ICD‐11 (Sellbom et al. 2024); however, the present study will focus on the self‐report form.

The popularity of the PDS‐ICD‐11 is reflected in a growing body of validation studies, which have examined its utility across a range of community and clinical samples. Building upon existing PD severity measures, the PDS‐ICD‐11 is unique in its use of a 5‐point bipolar scale to assess the eight areas of self‐ and interpersonal functioning along with two areas of emotional and behavioural manifestations. The middle response score of zero reflects healthy personality functioning, with dysfunction represented in opposing directions at either of the poles (e.g., low vs. high self‐worth, behavioural under‐control vs. over‐control). Bach et al. (2021) initially validated the PDS‐ICD‐11 in both a community (United States) and small clinical (New Zealand) sample, supporting its structural validity as capturing a unidimensional latent construct indicative of ICD‐11 PD severity. However, low endorsement within the sample for the item assessing harm to others resulted in a low factor loading of this item onto ICD‐11 PD severity. Total scores on the PDS‐ICD‐11 also demonstrated convergent validity with other measures of personality impairment, in particular the Level of Personality Functioning Scale—Brief Form (LPFS‐BF 2.0; Weekers et al. 2019), Standardised Assessment of Severity of Personality Disorders (SASPD; Olajide et al. 2018) and a latent severity score of the Personality Diagnostic Questionnaire‐4 (PDQ‐4; Hyler 1994).

Following this initial validation, Zimmermann et al. (2023) developed a German version of the PDS‐ICD‐11 and used a community sample to examine structural and convergent validity. Their findings aligned with those of the English version, supporting a unidimensional structure and exhibiting strong associations with measures of impairment and domain scores. Uniquely, nominal response models were used to confirm the bipolarity of items in assessing dysfunction, finding appropriate support for this scoring scheme. Harm to others once again exhibited low levels of endorsement and hence was the poorest indicator of ICD‐11 PD severity. Similar findings were observed in the Norwegian validation of the PDS‐ICD‐11 (Lorentzen et al. 2024), demonstrating a good model fit for a unidimensional model and a low factor loading for the item assessing harm to others. However, the validation of the Spanish PDS‐ICD‐11 indicated a satisfactory loading of harm to others onto the severity dimension, potentially due to the use of a mixed clinical and community sample (Gutiérrez et al. 2023).

Corroborating these findings, Bach et al. (2023) validated the Danish version of the PDS‐ICD‐11 in a community sample, supporting its unidimensionality and correlations with poor quality of life, impairments in personality, social and occupational functioning. Consistent with previous findings in community samples, the item assessing harm to others was endorsed at a low rate, resulting in a low factor loading. A further aim of this study was to determine normative‐based cut‐off scores, with the authors concluding that scores of 12, 16 and 19 likely correspond to Mild PD, Moderate PD and Severe PD, respectively, and scores between 9 and 12 likely indicate subthreshold personality difficulty.

The Spanish version of the PDS‐ICD‐11 has been further normed and validated for use in an adolescent Peruvian community sample (Hualparuca‐Olivera et al. 2024). Contrasting results in adult samples, a two‐factor structure was supported over the unidimensional model, thought to mirror internalising–externalising domains. Substantial correlations between the PDS‐ICD‐11 and external measures of personality pathology were demonstrated, with total scores showing stronger associations with internalising than with externalising psychopathology.

Clinical validations of the PDS‐ICD‐11 have supported its use as a diagnostic screening measure. The initial validation of the PDS‐ICD‐11 established its ability to differentiate between the presence and absence of a PD diagnosis in a small New Zealand community mental health sample, identifying a cut‐score of 17.5 as a threshold at which further clinical investigation into the possibility of a PD may be warranted (Bach et al. 2021). Expanding on this, Brown and Sellbom (2023) sought to further establish the construct validity of the PDS‐ICD‐11 for clinical populations, using a large New Zealand community mental health sample. Within this sample, the PDS‐ICD‐11 was able to differentiate between levels of personality dysfunction, demonstrated moderate to large associations with clinician and informant ratings of personality dysfunction and recommended a revised cut‐score of 14 given concerns that a cut‐score of 17.5 was too conservative. Of note, total scores demonstrated stronger associations with external measures of general severity and internalising personality pathology, whereas correlations with externalising criteria were comparatively weaker. This finding potentially reflects the internalising profile of the sample but also raises the possibility that the PDS‐ICD‐11 is more sensitive to internalising personality pathology.

Indeed, concerns exist surrounding the indication that the total PDS‐ICD‐11 score may better reflect internalising rather than externalising forms of personality dysfunction (Bach et al. 2021; Brown and Sellbom 2023; Zimmermann et al. 2023). There remains a paucity of research surrounding the application of the PDS‐ICD‐11 to samples with higher externalising symptoms and higher proportions of severe personality dysfunction (Zimmermann et al. 2023), raising questions on its utility in clinical and forensic samples. Given the overrepresentation of severe PDs in incarcerated (Bulten et al. 2009; Ogloff et al. 2015) and substance use treatment populations (Köck and Walter 2018), and the emphasis of the ICD‐11 on severity of personality dysfunction as the sole diagnostic criterion, validating the PDS‐ICD‐11 in cohorts with higher rates of externalising symptoms is critical for establishing its utility across clinical and forensic settings.

The present study therefore aimed to validate the PDS‐ICD‐11 for use in a mixed sample of adults in prison or a rehabilitation program with co‐occurring substance use dependency and personality pathology. Firstly, we aimed to establish the structural validity of the PDS‐ICD‐11 through replicating its unidimensional structure. We then sought to identify associations between the PDS‐ICD‐11 and other self‐reported measures of personality dysfunction and severity, specifically the LPFS‐BF, the World Health Organisation 5‐item Well‐Being Index (WHO‐5), the Symptom Checklist 90 Revised (SCL‐90‐R), Personality Severity Index (PSI), and the Personality Inventory for DSM‐5 Short Form (PID‐5‐SF) total score (proxy for ICD‐11 PD/AMPD severity) and trait domain scores. Finally, we built upon existing literature supporting the PDS‐ICD‐11's construct validity by evaluating the total score against the schema mode model of personality pathology as per the Schema Mode Inventory ‐ Healthy Adult subscale (SMI; Lobbestael et al. 2010). We expected large correlations between total PDS‐ICD‐11 and all external measures of personality functioning. Further, we anticipated scores to be similar across both internalising and externalising measures.

## 1 Method

### 1.1 Participants and Procedure

The present study is based on a mixed sample (*N* = 233; 185 males; M_age_ = 37.35; SD_age_ = 10.95) of individuals in treatment for co‐occurring personality pathology and substance use dependency in Denmark, including 101 incarcerated adults (100% males; M_age_ = 34.57, SD_age_ = 9.27) and 132 rehabilitation patients (64% males; M_age_ = 39.47, SD_age_ = 11.60). As evident from Table 1, both groups exhibited signs of significant personality pathology, and average PDS‐ICD‐11 scores indicated moderate levels of personality dysfunction. The rehabilitation patient group reported higher levels of personality dysfunction (M = 13.34, SD = 5.89) than the incarcerated group (M = 10.50, SD = 5.24). Across both samples, Disinhibition and Negative Affectivity emerged as the most prominent domains, in that order. On average, the rehabilitation sample scored higher on Detachment, whereas the incarcerated sample scored higher on Antagonism.

**TABLE 1:** Descriptive characteristics and association with external criterion variables.

	Total sample					Incarcerated		Rehabilitation	
	M	SD	Range	γ₁	κ	M	SD	M	SD
PDS‐ICD‐11	12.11	5.78	26	0.26	−0.38	10.50	5.24	13.34	5.89
LPFS‐BF	26.53	7.31	33	0.15	−0.60	24.49	6.73	28.14	7.36
PSI	1.15	0.78	3.74	0.80	−0.05	0.95	0.72	1.30	0.79
WHO‐5	39.18	24.77	100	0.35	−0.68	48.44	24.13	32.09	22.92
SMI healthy adult	3.96	0.90	4.40	−0.05	−0.79	4.36	0.81	3.63	0.84
PID‐5‐SF severity	1.03	0.46	2.23	0.10	−0.51	0.93	0.45	1.11	0.46
Negative affectivity	1.23	0.70	2.83	0.30	−0.71	0.97	0.61	1.43	0.70
Detachment	1.02	0.69	2.67	0.30	−0.88	0.76	0.61	1.23	0.69
Antagonism	0.76	0.63	2.92	0.96	0.45	0.85	0.69	0.68	0.57
Disinhibition	1.34	0.70	2.92	−0.10	−0.77	1.19	0.67	1.45	0.70
Psychoticism	0.67	0.57	3.00	1.04	0.85	0.57	0.53	0.75	0.59

Abbreviations: LPFS‐BF = Level of Personality Functioning—Brief Form; PDS‐ICD‐11 = Personality Disorder Severity ICD‐11; PID‐5‐SF = Personality Inventory for DSM‐5 Short Form; PSI = Personality Severity Index, which is based on the mean of Symptom Checklist 90 Revised (SCL‐90‐R) subscale scores for Interpersonal Sensitivity, Hostility and Paranoid Ideation (Karterud et al. 1995); SMI = Schema Mode Inventory ‐ Healthy Adult subscale; γ₁ = Skewness; κ = Kurtosis.

Educational levels for the rehabilitants included 42.4% who had only completed primary school, 25% who had completed vocational training, 6% who had only graduated from high school, 20.5% who had completed a BA‐level education and 6.1% who had completed a MA‐level education. For the incarcerated adults, 44.6% had only completed primary school, 35.5% had completed vocational training, 11.9% had only graduated from high school, 6.9% had finished a BA‐level education and 1% had a MA‐level education. A total of 42.2% of the rehabilitants were in a relationship, while 46.5% of the incarcerated adults were in a relationship.

The two treatment settings were specialised in the assessment and treatment of personality pathology and related substance use. A total of 71% of the incarcerated adults and 88% of the rehabilitation patients had previously used mental health services, including completed previous rehabilitation programs for substance abuse. The rehabilitants were primarily addicted to alcohol (36%), central stimulants (34%), cannabis (18%), opioids (7%) and benzodiazepines (3%). The incarcerated adults were primarily addicted to central stimulants (51%), cannabis (28%), alcohol (13%), opioids (5%) and benzodiazepines (4%), in that order.

The rehabilitants were overall characterised by reduced occupational functioning, with 16.7% who were granted disability pension, compared to 8.9% in the incarcerated sample, indicating relatively higher occupational functioning.

All participants were consecutively enrolled in the study as a part of their routine clinical intake assessment and treatment program. Each participant was administered a battery of computerised self‐report inventories including the measures employed in the current study. As a part of this procedure, each participant has given their consent to have their anonymised data used for quality assurance and research purposes. The study was carried out according to the Danish Data Protection Agency and General Data Protection Regulation (GDPR).

### 1.2 Measures

#### 1.2.1 Personality Disorder Severity Scale ICD‐11 (PDS‐ICD‐11)

The PDS‐ICD‐11 is a 14‐item self‐report measure of personality dysfunction as defined by self and interpersonal dysfunction, emotional, cognitive and behavioural manifestations, and psychosocial impairment/global distress (Bach et al. 2021). Respondents are asked to select the description that best fits their level of functioning, with the first 10 items being bipolar and the final four items being unipolar. Responses are summed to a total score ranging from 0 to 32, where greater scores indicate greater severity of impairment. The Danish version of the PDS‐ICD‐11 was used in this study and was translated and back‐translated in the initial development of the English PDS‐ICD‐11 (Bach et al. 2021). Danish norms for the PDS‐ICD‐11 based on a community sample stipulate total scores of 12, 16 and 19 indicate mild, moderate and severe PD, respectively (Bach et al. 2023).

#### 1.2.2 Level of Personality Functioning Scale—Brief Form (LPFS‐BF 2.0)

The LPFS‐BF 2.0 is a 12‐item self‐report scale assessing PD severity according to the AMPD (Weekers et al. 2019). Respondents rate each item on a 4‐point Likert scale (*very false or often false*–*very true or often true* [1–4]), and items are summed to create a total score. Higher scores indicate higher degree of impairment in personality functioning. The LPFS‐BF has been validated for use in a range of international samples (Bach and Hutsebaut 2018; Natoli et al. 2022; Weekers et al. 2019, 2022) and aligns with other instruments measuring PD severity (Zimmermann et al. 2020). The present study used the Danish translation of the LPFS‐BF 2.0, which has established validity and reliability (Bach and Hutsebaut 2018). Internal consistency for the LPFS‐BF total scale was excellent in the current sample (α = 0.89).

#### 1.2.3 World Health Organisation 5 Well‐Being Index (WHO‐5)

The WHO‐5 is a 5‐item self‐report measure of subjective psychological well‐being (Bech 2012). Respondents are asked to rate each item on a 6‐point Likert scale (*not present*–*constantly present* [0–5]) with raw scores ranging from 0 to 25. Scores aim to capture how psychological disorders impair well‐being and quality of life, with higher scores indicating greater well‐being. Raw scores are multiplied by 4 to represent a 100‐point scale, with 0 indicating *worst thinkable well‐being* and 100 indicating *best thinkable well‐being*. For this study, the Danish version of the WHO‐5 (Bech 2012) was used as a measure of psychosocial functioning. The alpha coefficient for the WHO‐5 in the current study was 0.87.

#### 1.2.4 Symptom Checklist 90 Revised (SCL‐90‐R) ‐ Personality Severity Index

The SCL‐90‐R is a 90‐item self‐report scale assessing a variety of symptom distress (Derogatis 1992). Respondents rate each item on a 5‐point Likert scale (*not at all*–*extremely* [0–4]) where higher scores indicate greater distress. For this study, a Personality Severity Index (PSI) was calculated by averaging scores for interpersonal sensitivity, hostility and paranoid ideation subscales. The PSI was used to indicate personality impairment in participants, in line with previous literature (Karterud et al. 1995). The Danish version of the SCL‐90‐R exhibits good psychometric features (Olsen et al. 2004) and the PSI scale demonstrated excellent internal consistency in the current sample (α = 0.93).

#### 1.2.5 Personality Inventory for DSM‐5 Short Form (PID‐5‐SF)

The PID‐5‐SF (Maples et al. 2015) is an abbreviated 100‐item version of the original 220‐item PID‐5 (Krueger et al. 2012). Respondents rate each item on a 4‐point Likert scale (*very false or often false*–*very true or often true* [0–3]) according to how much they agree that each statement represents themselves. The PID‐5‐SF comprises 25 trait facets, which load on five higher‐order domains of Negative Affectivity, Detachment, Antagonism, Disinhibition and Psychoticism (Krueger et al. 2012), with the current study also using the total PID‐5‐SF score as a proxy for ICD‐11 PD/AMPD severity. The Danish version of the PID‐5‐SF has established reliability and validity (Bach et al. 2016). In the present study, alpha coefficients were acceptable for all five domains: Negative Affectivity (0.78), Detachment (0.80), Antagonism (0.82), Disinhibition (0.78) and Psychoticism (0.76). The PID‐5‐SF composite score, serving as proxy of ICD‐11 PD/AMPD severity, demonstrated excellent internal consistency (α = 0.97).

#### 1.2.6 SMI—Healthy Adult Mode Scale

The SMI Healthy Adult mode scale is a 10‐item self‐report measure of healthy adult functioning, which involves being mature, integrative and reflective while having a reality‐based view of oneself, reflecting accurate and beneficent self‐awareness. Such features of healthy functioning help maintain a stable sense of positive self‐worth and self‐coherence and facilitate effective self‐direction (Rafaeli and Shuv‐Ami 2025). The construct, including its SMI operationalisation, was originally defined and developed by Young and First (2003) in order to describe intact or uncompromised aspects of the person's core part (or lack thereof) in individuals with severe personality pathology (Lobbestael et al. 2010). Thus, improving healthy adult functioning is a central target of psychosocial interventions for PD as this healthy core is theorised to regulate and integrate other dysfunctional parts/modes of the person (Bach and Bernstein 2019). The healthy adult mode scale is therefore also anticipated to align with healthy personality functioning (i.e., being negatively correlated with the PDS‐ICD‐11 score) as an indication of construct validity. The Danish translation of the SMI, including the healthy adult scale, demonstrates good psychometric properties. The SMI Healthy Adult score's internal consistency was excellent in the current study (α = 0.88).

### 1.3 Data Analysis

All statistical analyses were conducted using R Studio v2024.12.0 + 467 and IBM SPSS Statistics v29. Descriptive statistics were calculated across the total sample and stratified by participant group (rehabilitation vs. incarcerated). Given recent findings supporting a two‐factor structure of the PDS‐ICD‐11 (Hualparuca‐Olivera et al. 2024), an exploratory factor analysis (EFA) was first conducted to evaluate the optimal structure in the current sample. The optimal factor extraction was assessed using objective parallel analysis and subjective scree‐plot inspection; confirmatory factor analysis was used to assess correspondence across studies. To establish criterion validity, bivariate correlations were conducted between PDS‐ICD‐11 total scores and external measures of personality functioning, with Fisher's z‐tests used to compare correlational magnitudes across groups. Danish norms for the PDS‐ICD‐11 were then used to characterise ICD‐11 PD severity across both samples.

Relevant assumption checks pertaining to linearity, normality and outliers were performed on the data. All assumptions were reasonably met, with Skewness and Kurtosis values found in Table 1. Minor deviations from linearity and outliers were observed via visual inspection of Q‐Q and box plots; however, these occurred only in the very extreme ranges of the distribution. Missing data were present on the PID‐5 subscales (*N* = 3; 1.3%), LPFS‐BF total score (*N* = 3; 1.3%) and the SMI Healthy Adult subscale (*N* = 15; 6.4%); as the proportion of missing data was very small, pairwise deletion was used to account for missing data.

## 2 Results

### 2.1 Descriptive Statistics

As displayed in Table 1, across the total sample, there were notable internalising and externalising features. Mean scores on the LPFS‐BF domains of negative affectivity and detachment suggest moderate internalising personality dysfunction, whereas average scores on disinhibition and antagonism (which were similar to negative affectivity) reflect externalising personality dysfunction in the sample. Of note, the incarcerated adult sample showed higher antagonism scores compared to rehabilitation patients, reflecting the antisocial nature of this sample. However, the rehabilitation sample showed higher average PDS‐ICD‐11 and LPFS‐BF 2.0 scores relative to prisoners, indicating higher levels of personality‐related impairment and distress. Table 1 displays total sample means, standard deviations and ranges for each criterion variable, alongside means and standard deviations separated by participant group.

### 2.2 Structural Validity

In terms of the EFA, a parallel analysis suggested a one‐factor solution as most appropriate based on comparison of eigenvalues with eigenvalues calculated from 1000 randomly generated samples (see Table S1). Moreover, a Scree‐plot analysis also clearly indicated a one‐factor model as the most optimal solution (see Figure S1). The model fit for a unidimensional CFA model was also acceptable (χ^2^ = 122.53 [df = 77, *p* < 0.001], CFI = 0.96, TLI = 0.96, SRMR = 0.057 and RMSEA = 0.050), which is largely consistent with previous international findings. McDonald's omega was 0.83, further supporting the unidimensionality of the PDS‐ICD‐11. As shown in Table 2, the median CFA loading coefficient was 0.59, ranging from 0.32 (‘harm to others’) to 0.72 (‘self‐worth’), which overall corresponds to the composition of loading patterns found in previous studies (see Table 2). For item 13 (‘harm to others’), only nine respondents endorsed the most severe level of harm. The internal consistency for the PDS‐ICD‐11 scale score was acceptable in terms of a Cronbach's alpha coefficient of 0.83.

**TABLE 2:** Unidimensional factor loadings based on standardised CFA.

PDS‐ICD‐11 item	Present study	Comparison with other studies				
		Danish community	United States	Germany	Spain	Peru 10–17 years
**1. Identity**	0.63	0.44	0.73	0.82	0.69	0.98
**2. Self‐worth**	0.72	0.78	0.83	0.81	0.82	0.76
**3. Self‐perception**	0.65	0.55	0.55	0.65	0.73	0.71
**4. Goals**	0.59	0.68	0.73	0.67	0.68	0.86
**5. Interest in relationships**	0.53	0.72	0.72	0.64	0.73	0.68
**6. Perspective taking**	0.54	0.67	0.60	0.57	0.64	0.68
**7. Mutuality in relationships**	0.67	0.75	0.70	0.66	0.80	0.78
**8. Disagreement management**	0.50	0.62	0.66	0.66	0.68	0.84
**9. Emotion regulation**	0.51	0.65	0.71	0.65	0.83	0.79
**10. Behavioural control**	0.58	0.70	0.76	0.68	0.76	0.63
**11. Reality testing**	0.64	0.66	0.55	0.60	0.59	0.71
**12. Harm to self**	0.58	0.47	0.59	0.54	0.66	0.57
**13. Harm to others**	0.32	0.28	0.42	0.24	0.50	0.62
**14. Psychosocial impairment**	0.69	0.44	0.73	0.82	0.69	0.84

Abbreviation: CFA, confirmatory factor analysis.

### 2.3 Associations With Criterion Variables

To examine the criterion validity of the PDS‐ICD‐11, we calculated correlations between PDS‐ICD‐11 total scores and established self‐reported measures of personality dysfunction and severity. As seen in Table 3, most associations with criterion variables were large (*r* ≥ 0.50) across the total sample and when separating the sample into rehabilitation and incarcerated groups. To test for statistically significant differences in correlations between groups, we calculated Fisher's z‐tests (Fisher 1921). From this perspective, one criterion correlation significantly differed between groups: Antagonism (incarcerated *r* = 0.55, rehabilitation *r* = 0.26, *z* = 2.07, *p* = 0.038). The strongest associations were observed with the LPFS‐BF, the PID‐5 SF severity score and the SCL‐90‐R PSI index, providing convergent evidence for the validity of the PDS‐ICD‐11. Although total scores showed strong correlations with internalising‐related indicators (i.e., LPFS‐BF), similar associations were observed with externalising‐related indicators. Specifically, total PDS‐ICD‐11 scores were highly correlated with Disinhibition in both groups (*r* = 0.60) and with Antagonism in the incarcerated sample (*r* = 0.55). Large correlations were also observed between total scores and the PSI (*r* = 0.72), which includes externalising features of hostility and interpersonal sensitivity.

**TABLE 3:** Association of PDS‐ICD‐11 with external criterion variables by group.

	Total sample	Incarcerated	Rehabilitation	Fisher's z‐test	
	*r*	*r*	*r*	*z*	*p*
LPFS‐BF	0.79	0.76	0.79	−0.56	0.575
PSI	0.72	0.79	0.70	1.52	0.128
WHO‐5	−0.54	−0.49	−0.51	0.20	0.842
SMI healthy adult	−0.70	−0.65	−0.68	0.40	0.688
PID‐5‐SF severity	0.74	0.79	0.70	1.52	0.128
Negative affectivity	0.63	0.55	0.62	−0.80	0.426
Detachment	0.59	0.63	0.51	1.33	0.182
Antagonism	0.34	0.55	0.26	2.63	0.009
Disinhibition	0.60	0.62	0.55	0.80	0.426
Psychoticism	0.54	0.48	0.55	−0.72	0.477

Abbreviations: LPFS‐BF = Level of Personality Functioning—Brief Form; PID‐5‐SF = Personality Inventory for DSM‐5 Short Form; PSI = Personality Severity Index, which is based on the mean of Symptom Checklist 90 Revised (SCL‐90‐R) subscale scores for Interpersonal Sensitivity, Hostility and Paranoid Ideation (Karterud et al. 1995); SMI = Schema Mode Inventory ‐ Healthy Adult scale.

### 2.4 Distribution of ICD‐11 PD Severity Levels

To characterise the severity of personality dysfunction in the present sample, Danish norms for the PDS‐ICD‐11 (Bach et al. 2023) were used to estimate rates of tentative ICD‐11 PD severity levels. As seen in Table 4, the majority of the total sample (52.3%) may likely have met the requirements for a PD as evidenced by at least mild dysfunction. Only a minority of participants (28.8%) did not meet the indicated threshold requirement for PD, consistent with the fact that participants were recruited from services specialising in personality pathology. Notably, the rehabilitation sample had a larger proportion of participants with self‐reported PD‐level dysfunction (60.6%) than the incarcerated sample (41.5%), and 25% of the rehabilitation sample reported a level of dysfunction corresponding to severe PD compared to only 5.9% of the incarcerated sample.

**TABLE 4:** Distribution of severity levels in the total sample and subsamples.

	Total (*N* = 233)	Rehabilitation (*n* = 132)	Incarcerated (*n* = 101)
None	67 (28.8%)	28 (21.2%)	39 (38.6%)
Personality difficulty	44 (18.9%)	24 (18.2%)	20 (19.8%)
Mild–moderate dysfunction	59 (25.3%)	32 (24.2%)	27 (26.7%)
Moderate–severe dysfunction	24 (10.3%)	15 (11.4%)	9 (8.9%)
Severe–extreme dysfunction	39 (16.7%)	33 (25.0%)	6 (5.9%)

*Note:* Based on empirically informed cut‐offs derived from the Danish general male population (Bach et al. 2023).

## 3 Discussion

The present study aimed to further validate the PDS‐ICD‐11 for use in a Danish mixed sample of adult inmates and rehabilitation patients in treatment for co‐occurring substance use dependency and personality pathology. We aimed to establish the structural validity of the PDS‐ICD‐11 and replicate its unidimensional structure, identify associations with conceptually relevant extra‐test variables and examine scores across internalising and externalising measures. Taken together, the findings of the present study support the use of the PDS‐ICD‐11 in externalising forensic and rehabilitation patient samples. Specifically, the results indicated support for the unidimensional structure of the PDS‐ICD‐11 and positive support for construct validity, upon which we elaborate further.

First, we found that all self‐reported PDS‐ICD‐11 items fitted a unidimensional model. These findings are largely consistent with previous research that has supported a one‐factor structure for the PDS‐ICD‐11 (Bach et al. 2021; Bach et al. 2023; Brown and Sellbom 2023; Gutiérrez et al. 2023; Lorentzen et al. 2024; Zimmermann et al. 2023). However, this finding contrasted with those of Hualparuca‐Olivera et al. (2024), who concluded that a two‐factor model demonstrated the best fit in an adolescent sample. Differing expressions of personality dysfunction in adolescent samples may explain this divergence in findings; indeed, identity disturbances, self‐harm and harm to others were endorsed at substantially higher rates in Hualparuca‐Olivera et al.'s (2024) sample compared to the current sample, which aligns with current conceptualisations of adolescent personality pathology that emphasise greater instances of self‐harm and externalising personality features in adolescents with PDs (Chanen et al. 2022).

Despite the externalising nature of the sample, the item assessing harm to others was endorsed at a low rate and consequently demonstrated a low factor loading. This finding aligns with previous research conducted in community and internalising clinical samples (Bach et al. 2021; Bach et al. 2023; Lorentzen et al. 2024; Zimmermann et al. 2023) in which similar patterns of low endorsement and low factor loadings have been reported. However, it contrasts previous research in a mixed clinical and community sample characterised by more externalising features (Gutiérrez et al. 2023) in which a higher endorsement of this item might have been expected. Given the prominence of externalising traits such as antagonism in our sample of incarcerated adults, and the higher ICD‐11 PD severity observed across both samples, the low endorsement and loading of this item was somewhat unexpected. One possible explanation is that externalising features do not tend to cause significant impairment and distress and therefore may not be perceived by respondents as indicative of dysfunction. Alternatively, as only severe personality manifestations tend to be associated with harm directed to others, and are often more present in a prison context, such severe presentations may have been underrepresented in our incarcerated sample. This could reflect a broader sampling issue, whereby individuals with more pronounced personality pathology did not engage in the treatment programs from which our sample was drawn. Furthermore, low endorsement of socially undesirable features such as harm to others may reflect the limitations of self‐report measures in this context, highlighting the value of clinician ratings in assessing socially undesirable externalising features of personality dysfunction.

Second, the PDS‐ICD‐11 demonstrated strong criterion validity, with total scores correlating most strongly with external measures of personality impairment, aligning with previous findings (Brown and Sellbom 2023; Lorentzen et al. 2024). Contrasting previous literature (Bach et al. 2023; Brown and Sellbom 2023; Zimmermann et al. 2023), we found that total PDS‐ICD‐11 scores were similarly correlated with both internalising and externalising personality dysfunction. One possible explanation for this discrepancy is that prior studies have predominantly drawn from community and clinical populations with a higher prevalence of internalising psychopathology in which overall personality dysfunction may therefore have been more strongly driven by internalising processes. In contrast, our mixed sample, especially the incarcerated adults, endorsed externalising and internalising traits at similar levels. This pattern is consistent with findings from Hualparuca‐Olivera et al. (2024), who observed a similar trend in adolescents characterised by greater externalising personality dysfunction. Accordingly, in samples where personality dysfunction is thought to arise from both internalising and externalising dysfunction, total PDS‐ICD‐11 scores may correlate similarly across both domains.

Our subgroup analyses further supported these interpretations. Whereas both samples displayed similar patterns of associations with external criterion measures, the incarcerated sample showed stronger magnitude correlations with PDS‐ICD‐11 total scores and antagonism. Despite higher levels of externalising traits and greater expected antisocial behaviour, incarcerated individuals exhibited lower overall ICD‐11 PD severity. This finding may reflect differences in insight or a reluctance to endorse harm to others on self‐report measures. Alternatively, it may relate to differences in recruitment or criteria for inclusion in the treatment programs from which these samples were drawn. Ultimately, these findings suggest that the predominant presentation of personality dysfunction within a given sample may influence the external correlates of PDS‐ICD‐11 scores.

In terms of implications, the current study adds support to the utility of the PDS‐ICD‐11 as a screening measure for ICD‐11 PD severity, which might be employed as a tool for further dialogue with inmates and rehabilitants about their personality‐related resources and risk factors, and for clinicians to decide whether a diagnostic interview should be carried out to establish the presence of a PD diagnosis. Importantly, the current findings bolster those of Hualparuca‐Olivera et al. (2024) that scores on this inventory are also related to externalising dysfunction in samples in which such is likely to be present. This overall finding is important to researchers and clinicians who use the PDS‐ICD‐11 in forensic, correctional and other clinical settings in which individuals' personality dysfunction might manifest in a more externalising manner.

The present study was not without limitations. First, we relied exclusively on self‐report instruments, which may inflate correlations between instruments due to mono‐method bias. The use of self‐report could be potentially problematic in PDs and incarcerated samples, which tend to be characterised by denial, social desirability and overall inaccurate self‐perception, which may have contributed to the low endorsement of harm to others and overall lower ICD‐11 PD severity in the incarcerated group. Furthermore, the endorsement of dissocial responses (e.g., harm to others) may carry immediate consequences within forensic settings and may have contributed to a reluctance to answer such self‐report questions within this context. Although the PDS‐ICD‐11 has been validated as a self‐report tool, future research should seek to employ multimethod designs incorporating clinician ratings (Sellbom et al. 2024), marker variables (Howard et al. 2024) or behavioural observations to provide external validation and more accurately assess socially undesirable or underreported symptoms.

Second, co‐occurring diagnoses could not be considered due to a lack of diagnostic characterisation of the sample. We were therefore unable to examine the discriminant validity of the PDS‐ICD‐11 against forms of impairment resulting from clinical disorders such as depression or anxiety. It was also difficult to determine the extent to which co‐occurring conditions may have contributed to elevated PDS‐ICD‐11 total scores. Without this clarity, isolating the unique contribution of personality pathology remains challenging. To strengthen the specificity and utility of the PDS‐ICD‐11 as a diagnostic tool, future research should incorporate thorough diagnostic assessments to better delineate the interplay between ICD‐11 personality dysfunction and co‐occurring mental health conditions.

Third, while our study used recommended thresholds from a representative Danish general population sample to classify ICD‐11 PD severity (Bach et al. 2023), the direct application of these thresholds to a clinical and incarcerated sample is likely to be imprecise. Given that rates of comorbidity and environmental stressors, particularly within prison settings, may influence the expression and severity of personality dysfunction, further research is needed to cross‐validate and potentially refine these thresholds in clinical and incarcerated populations. More specifically, future studies should aim to use clinical samples with reliably diagnosed PD to further elaborate on specific cut‐off scores for screening purposes in such populations.

Fourth, the incarcerated sample for this study consisted exclusively of men. Previous literature has indicated that women in incarcerated samples tend to exhibit greater PD severity and complexity of psychopathology, including greater borderline personality features (Falk et al. 2017). Accordingly, future studies should aim to assess the validity of the PDS‐ICD‐11 in capturing personality dysfunction in incarcerated women. Such investigations could be achieved via stratified sampling with mixed‐gender samples or through studies specifically targeting female prison populations. Conducting research with female incarcerated populations would help to clarify whether the PDS‐ICD‐11 performs equivalently across male and female populations.

Finally, it was not within the scope of the present study to examine the measurement invariance of the PDS‐ICD‐11 across clinical and incarcerated samples. Future research using larger and more diverse samples should therefore aim to examine the measurement invariance of the PDS‐ICD‐11 across different groups.

## Conflicts of Interest

The authors declare no conflicts of interest.

## Supporting information


**Table S1:** Parallel analysis.
**Figure S1:** Scree‐plot analysis.

## Data Availability

The data that support the findings of this study are available from the corresponding author upon reasonable request.
