# Supplementary material for: Validation of the Personality Disorder Severity for ICD‐11 (PDS‐ICD‐11) Scale in a Danish Mixed Clinical and Prison Treatment Sample
Source: Personal Ment Health. 2025 Sep 16;19(4):e70039. doi: 10.1002/pmh.70039 (PMC12440841; doi:10.1002/pmh.70039)

**Supplementary material**

**Table S1. Parallel analysis**

Based on parameters provided by the researcher, this engine calculates eigenvalues from randomly generated correlation matrices. These can then be compared with eigenvalues extracted from the researcher's dataset. The number of factors to retain will be the number of eigenvalues (generated from the researcher’s dataset) that are larger than the corresponding random eigenvalues (Horn 1965).

| Factor | Eigenvalues: current data | Eigenvalues: randomly generated correlation matrices |
| --- | --- | --- |
| 1 | 4,458 | 1,434 |
| 2 | 1,148 | 1,323 |
| 3 | 1,031 | 1,244 |
| 4 | 1,012 | 1.178 |

**Figure S1. Scree-plot analysis**


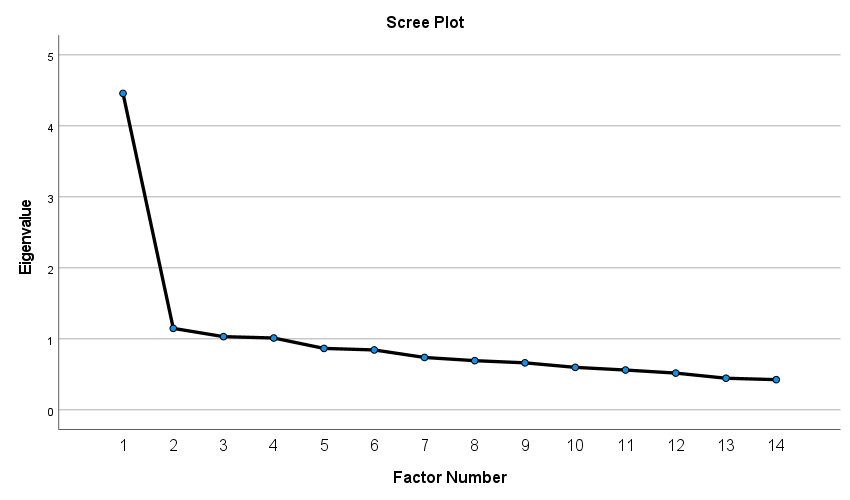

Supplement: Supplementary file 1 — Table S1: Parallel analysis. Figure S1: Scree‐plot analysis. [file PMH-19-0-s001.docx]
